# An International Study of Factors Affecting Variability of Dosimetry Calculations, Part 4: Impact of Fitting Functions in Estimated Absorbed Doses

**DOI:** 10.2967/jnumed.124.268612

**Published:** 2025-03

**Authors:** Sara Kurkowska, Julia Brosch-Lenz, Yuni K. Dewaraja, Eric Frey, John Sunderland, Carlos Uribe

**Affiliations:** 1 Department of Nuclear Medicine, Pomeranian Medical University, Szczecin, Poland;; 2 Department of Integrative Oncology, BC Cancer Research Institute, Vancouver, British Columbia, Canada;; 3 Institute of Nuclear Medicine, Glen Burnie, Maryland;; 4 Department of Radiology, University of Michigan, Ann Arbor, Michigan;; 5 Rapid, LLC, Baltimore, Maryland;; 6 Department of Radiology, Johns Hopkins University, Baltimore, Maryland;; 7 Department of Radiology, University of Iowa, Iowa City, Iowa;; 8 Department of Molecular Imaging and Therapy, BC Cancer, Vancouver, British Columbia, Canada; and; 9 Department of Radiology, University of British Columbia, Vancouver, British Columbia, Canada

**Keywords:** ^177^Lu, dosimetry, fitting and integration, radiopharmaceutical therapies, SPECT/CT

## Abstract

Individualized radiopharmaceutical therapies guided by patient-specific absorbed dose (AD) assessments using nuclear medicine imaging have the potential to improve both efficacy and safety. Understanding sources of variability in AD calculations is critical for standardization. The Society of Nuclear Medicine and Molecular Imaging Dosimetry Task Force launched the ^177^Lu Dosimetry Challenge to evaluate variability across steps within the dosimetry workflow. This work aimed to assess the variability in ADs due to different fitting and integration methods. **Methods:** Anonymized datasets from 2 patients treated with ^177^Lu-DOTATATE, including serial SPECT/CT scans, segmented organs and lesions, and time-integrated activity maps, were made available online. Participants were invited to perform dosimetry calculations and submit their results using standardized submission spreadsheets. Fitting approaches were categorized, and relative AD variability was analyzed using the quartile coefficient of dispersion and interquartile range. **Results:** The variability in AD due to the fitting step for patient A’s kidneys was less than 1%. In contrast, patient B’s kidneys showed higher variability, with values below 10%. Lesions exhibited more variability in fitting than did kidneys, with variability within 25%. **Conclusion:** The contribution of variability caused by fitting and integration is small for healthy organs. By following recommendations such as selection of appropriate functions, pharmacokinetic modeling, and sanity checks, this variability can be further reduced.

## Visual Abstract

**Figure:**
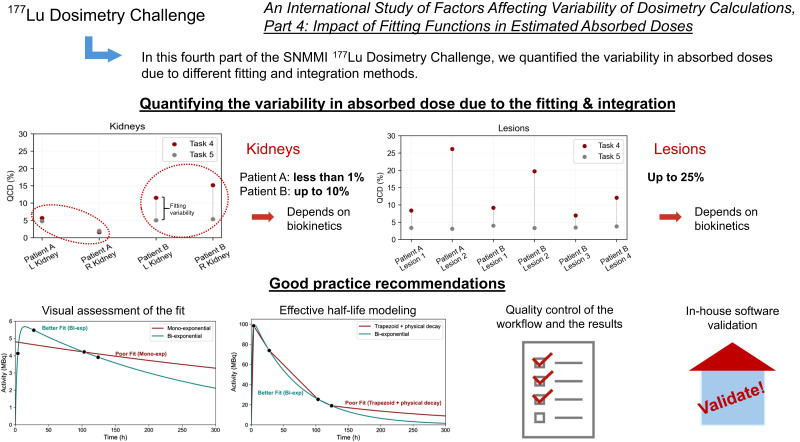

Radiopharmaceutical therapies (RPTs) are oncologic treatments that deliver radiation directly to cancer cells. Over the last decade, RPTs have shown great promise in managing neuroendocrine tumors (1) and prostate cancer (2). The efficacy and safety of RPTs highly depends on the absorbed dose (AD) delivered to the tumors and healthy organs, respectively. Accurate dosimetry calculations hold promise for optimal treatment planning (3,4) and verification after therapy. Although substantial progress has been made in the field, there is still work to be done to enable routine clinical adoption.

A key challenge in advancing RPTs is variability in AD calculations caused by a lack of standardization in internal dosimetry. This variability raises concerns about the reliability of dosimetry in enabling individualized treatments. A data-driven approach to understanding these sources of variability is essential for establishing standardized methods. To address this, the Society of Nuclear Medicine and Molecular Imaging (SNMMI) launched the ^177^Lu Dosimetry Challenge. This initiative aimed to evaluate variability across different steps of the dosimetry workflow through 5 distinct tasks, each isolating specific aspects of the process (5).

Numerous studies have explored the impact of different fitting functions or simplified approaches relying on fewer data points for time-integrated activity (TIA) (6–8). Studies such as the one by Zvereva et al. (9) suggested that TIA coefficients (TIA normalized by injected activity) have the greatest impact on the operator component of variation between AD estimates.

This study evaluates variability in the clinical setting, incorporating input from a diverse group of participants around the world working with the same well-curated dataset. Participant experience ranged from novice to expert, reflecting clinical diversity. This approach provides insights into real-world complexities associated with the handling of 4-point SPECT/CT image data, in which physicists are tasked with finding a fitting and integration approach that strikes a balance between simplicity (i.e., fewer fitting parameters) and accuracy.

The aim of this fourth installment of the SNMMI ^177^Lu Dosimetry Challenge was to quantify the variability in ADs due to different fitting and integration approaches that were reported by the participants across different institutions. On the basis of these data, we recommend harmonizing methods to reduce variability and highlight clinical challenges contributing to AD variability.

## MATERIALS AND METHODS

### Patient Data and Dosimetry Challenge Tasks

Two patients treated with ^177^Lu-DOTATATE (patient A was injected with 7.21 GBq and patient B was injected with 7.31 GBq) underwent a series of 4 SPECT/CT scans after therapy. Imaging was performed for patient A at 3.7, 27.7, 103.1, and 124.0 h and for patient B at 3.7, 32.6, 99.6, and 193.3 h after therapy administration. Quantitative SPECT images and metadata can be accessed at https://deepblue.lib.umich.edu/data (10–12). Participants were provided with images and volumes of interest (VOIs) in radiotherapy structure set (RTStruct) and binary mask formats and were asked to calculate the ADs to the kidneys (the VOIs included the medulla and cortex of the kidneys), spleen (patient A only), healthy liver, and specific tumors (lesions 1 and 2 in patient A and lesions 1–4 in patient B) and to report various variables and intermediate values relevant to the dosimetry workflow. More details about the patients and the challenge are provided in Uribe et al. (5).

The data were analyzed in 3 distinct steps. In step 1, the fitting methodology analysis, we collected and categorized the fitting approaches used by participants based on submitted data to explore the diversity of methods for fitting time–activity data. Fitting methods were identified across 3 time intervals: interval 1 (I1), from time 0 to the first (or second) time point; I2, from the first (or second) time point to the fourth time point; and I3, from the fourth time point to infinity.

In step 2, quantifying the relative variability in AD due to different fitting approaches, we assessed the relative variability in AD resulting from the implementation of different fitting approaches by comparing participant-reported AD results from tasks 4 and 5. In task 4, images were provided in Bq/mL, and VOIs for each region were defined. Participants were required to apply these VOIs to images to obtain activities, perform fitting and integration to obtain TIAs, and use the TIAs to calculate AD. In task 5, participants used a precalculated TIA image in (Bq/mL)·s generated by organizers through voxelwise fitting using the MIM MRT Dosimetry package (MIM Software, GE HealthCare), in which time–activity data at each voxel were fit using multiple exponential functions through standard least-squares optimization. For each voxel, the best-fitting function was automatically selected based on the Akaike Information Criterion, allowing different models across voxels to account for varying biokinetics. Participants applied the same set of VOIs as in task 4 to extract organ TIA, eliminating variability from fitting, calculating TIA, and focusing only on the methods for converting it to AD (13).

Here, we included only data from participants who completed both tasks and used the quartile coefficient of dispersion (QCD), defined as the difference between the 75th and the 25th quartiles divided by their sum, to quantify variability. The QCD was chosen because it is less sensitive to outliers in the data than the coefficient of variation. The outliers observed in this analysis are likely the result of errors, rather than variability inherent to the fitting process. Nevertheless, we report both the number and the percentage of outliers. Although these outliers may not be crucial for describing the fitting variability, they could still influence the dosimetry comparability of different centers. The difference in variability between tasks 4 and 5 represents the relative variability introduced by the fitting step.

Because the application of VOIs (either RTStruct or binary mask) and the calculation of ADs from TIAs involved different software and methods, the variability estimated with QCD also reflects the impact of these factors. To minimize their influence and focus on the fitting, we also conducted a paired analysis. In this analysis, for each participant, we computed the difference between ADs (ΔAD) from tasks 4 and 5, normalized by the mean AD in task 5 (${\bar{\mathrm{AD}}}_{\mathrm{task}5}$) as reported by all participants. ΔAD reflected the dose difference between participant-performed fitting and fitting provided in task 5. In task 5, the organizers used only 1 method to generate the TIA map, whereas in task 4, participants used various methods. The variability of $\Delta \mathrm{AD}/{\bar{\mathrm{AD}}}_{\mathrm{task}5}$, assessed using the interquartile range (IQR), reflects differences across different techniques. Outliers likely contribute to AD variability, and this insight informed some of our recommendations. Outliers are defined as data points lying outside the range of Q1−1.5 × IQR to Q3 + 1.5 × IQR, where Q1 and Q3 denote the first and third quartiles. We report both the number and the percentage of outliers.

In step 3, baseline fitting variability analysis, we aimed to quantify the impact of various fitting functions and approaches on TIA and AD variability. A single dosimetry expert conducted the analysis using the fitting methods identified in step 1, eliminating variability caused by differences in user decisions (e.g., initial parameter estimates, fitting software and algorithms, integration methods, and application of the provided VOIs to estimate activities). This step focuses on the variability stemming from the use of different fitting functions and integration methods, aiming to identify the approaches that may have contributed to the variability.

A baseline time–activity dataset was generated in MIM version 7.2.1 using the VOIs in RTStruct and binary mask formats provided in task 4. Using 11 fitting methods identified in step 1, we calculated TIAs with the SciPy Python library (14) for nonlinear least-squares fitting using the Levenberg–Marquardt algorithm. Because weighting methods were not specified by participants, we present unweighted fitting results in the Results section. However, we acknowledge the importance of weighting in the fitting procedure, and following the recommendations from Ivashchenko et al. (15), we implemented an error model for fitting functions described by 2 models: monoexponential and biexponential. The impact of the weighted fitting is discussed in the supplemental materials (supplemental materials are available at http://jnm.snmjournals.org), and weighted and unweighted fits for all regions are shown for patients A and B in Supplemental Figures 1 and 2, respectively. Lastly, we used MIRDcalc version 1.1 (University of Florida and Memorial Sloan Kettering Cancer Center) (16) to convert TIAs to ADs.

On the basis of the participant methods, 3 approaches were used for modeling data in I1: constant activity from time 0 to the second time point (with the first time point excluded), linear uptake from (0,0) to the first time point, or biokinetics modeling using exponential functions. In I2, biokinetics were modeled either with single or multiple exponential functions or with trapezoidal interpolation (lines connecting data points). For I3, the biokinetics extended the curve from I2, or if the trapezoidal approach was applied, the monoexponential curve was modeled using the last 3 time points or assumed physical decay after the last time point.

To reproduce the participants’ fitting and integration approaches, exponential functions were used. First, we used the following monoexponential function:

$$A\left(t\right)=Ae^{-\lambda t},$$ Eq. 1

where *t* is time, *A* is activity at time 0 and λ is the decay constant. Some users assumed physical decay after the last time point. In this case, Equation 1 can be modified to the following:

$$A\left(t\right)=A_{p4}e^{-\lambda_{\mathrm{physical}}(t-t_{p4})},$$ Eq. 2

for $t\geq t_{p4}$, where *A*_*p*4_ is the activity at time point 4 and λ_physical_ is the physical decay constant,

Next, we used the biexponential functions

$${A\left(t\right)=A(e}^{-\lambda_{1}t}-e^{-\lambda_{2}t}),$$ Eq. 3

and

$$A\left(t\right)={A_{1}e}^{-\lambda_{1}t}+A_{2}e^{-\lambda_{2}t},$$ Eq. 4

where λ_1_ and λ_2_ are decay constants of two different pharmacokinetic phases and *A*_1_ and *A*_2_ are corresponding activities. The biexponential function in Equation 3 ensures that the activity is zero at time 0. This reduction in the number of fitting parameters can help reduce overfitting and potentially make the model more robust and generalizable. It is not recommended to apply a 4-parameter model (Eq. 4) to fit 4 data points.

On the basis of the participant methods for Equation 1, we examined scenarios in which the decay constant was either constrained or not to be larger than the physical decay constant of ^177^Lu (i.e., *λ* > $\lambda_{\mathrm{physical}}$).

Finally, we evaluated the variability of the area under the curve for each of the 3 time intervals of the time–activity curve described earlier to the final TIA when different fitting methods were used.

## RESULTS

### Step 1: Fitting Methodology Analysis

**FIGURE 1.**
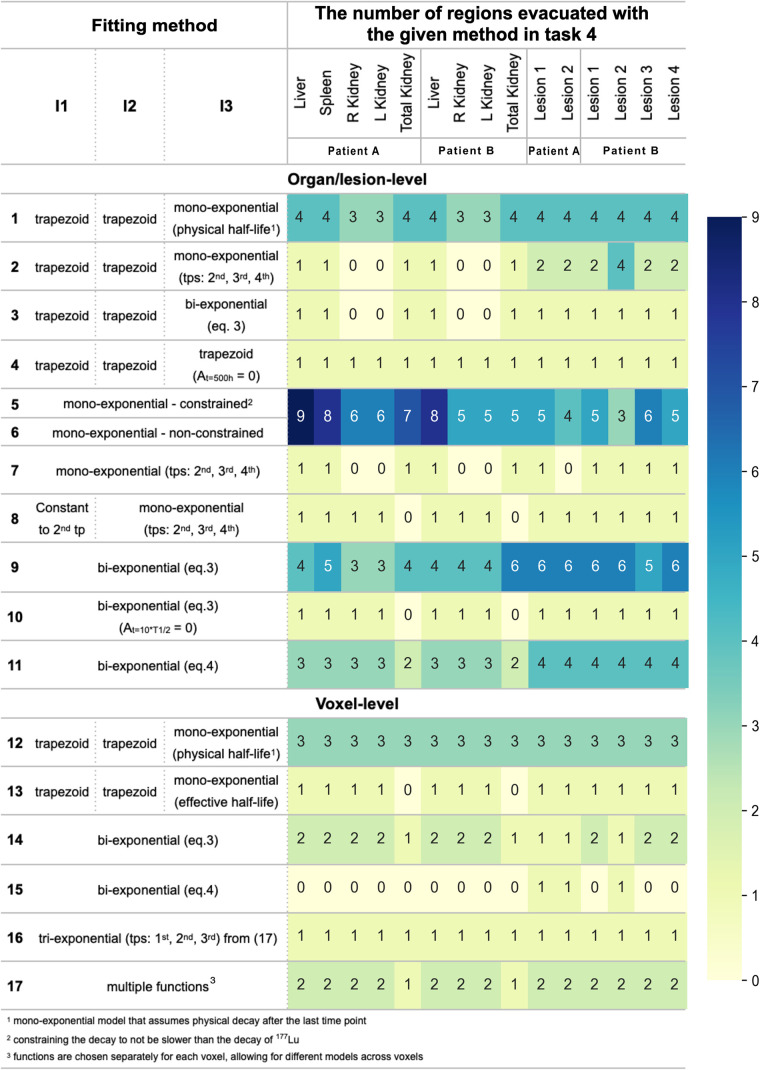
Heatmap of fitting methods used by participants in task 4 to model biokinetics across I1–I3 on both organ or lesion and voxel levels. Gradient shows usage frequency, with values annotated. All methods model 4 time points unless specified otherwise. T_1/2_ = half-life; tps = time points.

We analyzed 78 submissions from task 4 (39 per patient). Figure 1 summarizes the fitting methods used at the organ or lesion and voxel levels, illustrating the diversity of models and highlighting the regions where certain methods were preferred over others. The heatmap highlights the most used fitting methods. Method 16 uses a triexponential approach (17). Fitting was more frequently conducted at the organ or lesion level, rather than at the voxel level. Monoexponential and biexponential fits were most common for organs and lesions, respectively.

### Step 2: Quantifying the Relative Variability in AD Due to Different Fitting Approaches

**FIGURE 2.**
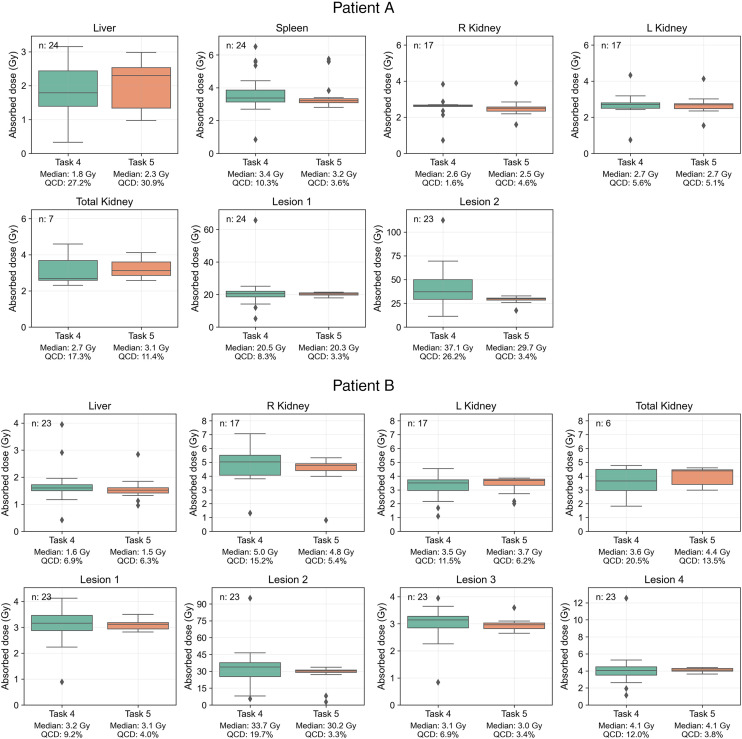
Distribution of participant-reported ADs for all regions, with median and QCD annotated for each box plot. Submission counts are shown at upper left.

**FIGURE 3.**
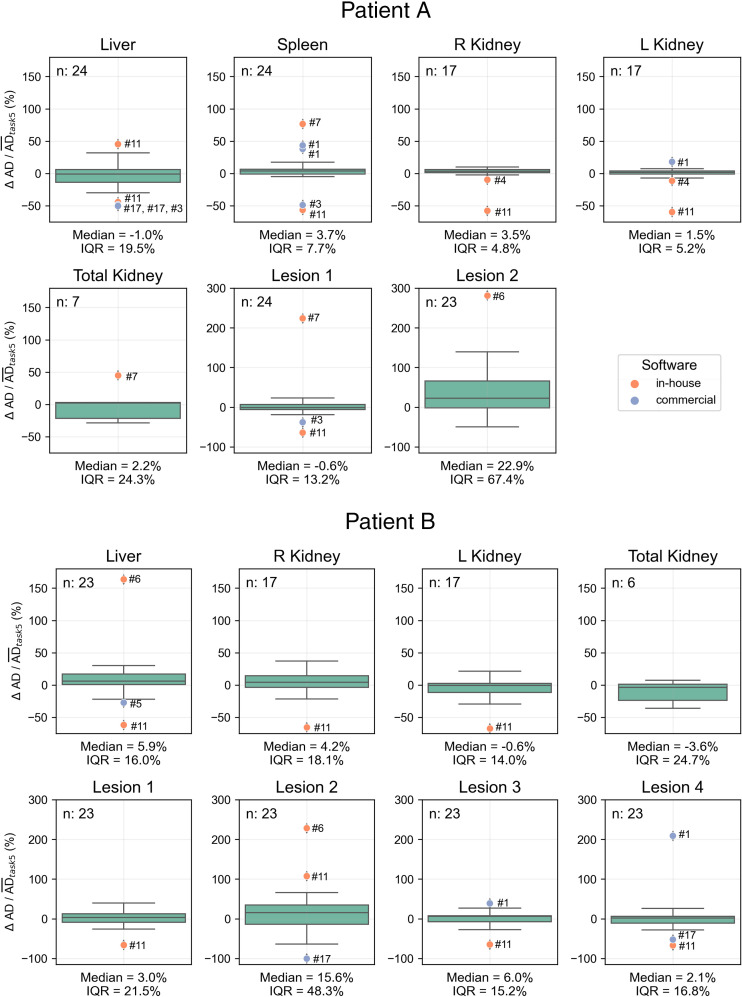
Distribution of $\Delta \mathrm{AD}/{\bar{\mathrm{AD}}}_{\mathrm{task}5}$ calculated for all regions using participant-reported ADs, with median and IQR annotated. Submission counts per region are shown at upper left. Outliers, labeled with fitting methods from Figure 1, are color-coded for in-house and commercial software implementations.

We analyzed 47 submissions from the participants in tasks 4 and 5 (24 for patient A and 23 for patient B). Figure 2 shows box plots of the ADs for each region reported in both tasks, with median and QCD annotated. Table 1 shows the difference in QCD between tasks. The QCD was higher for task 4 than for task 5 for all organs and lesions of both patients, except for the liver and right kidney of patient A. Even for these cases, AD ranges were broader in task 4. The largest differences were observed for lesion 2 of patient A and lesion 2 of patient B. For the kidneys, the variability was larger for patient B. ΔAD/${\bar{\mathrm{AD}}}_{\mathrm{task}5}$ are presented in Figure 3, with median and IQR annotated. Outliers, color-coded by in-house or commercial software implementation and labeled with fitting method numbers from Figure 1, were mostly linked to the same participants who systematically reported higher or lower values. Supplemental Table 1 summarizes outlier counts and percentages from Figures 2 and 3, showing more outliers in task 4 than in task 5 and more for patient A than for patient B.

**TABLE 1.** Difference in QCD Between Tasks 4 and 5 for Each Region

Region	Patient A (%)	Patient B (%)
Liver	−3.6	0.6
Spleen	6.7	
R kidney	−0.4	9.8
L kidney	0.9	6.5
Total kidney	5.9	7.0
Lesion 1	5.0	5.2
Lesion 2	23.1	16.5
Lesion 3		3.5
Lesion 4		8.3

### Step 3: Baseline Fitting Variability Analysis

**FIGURE 4.**
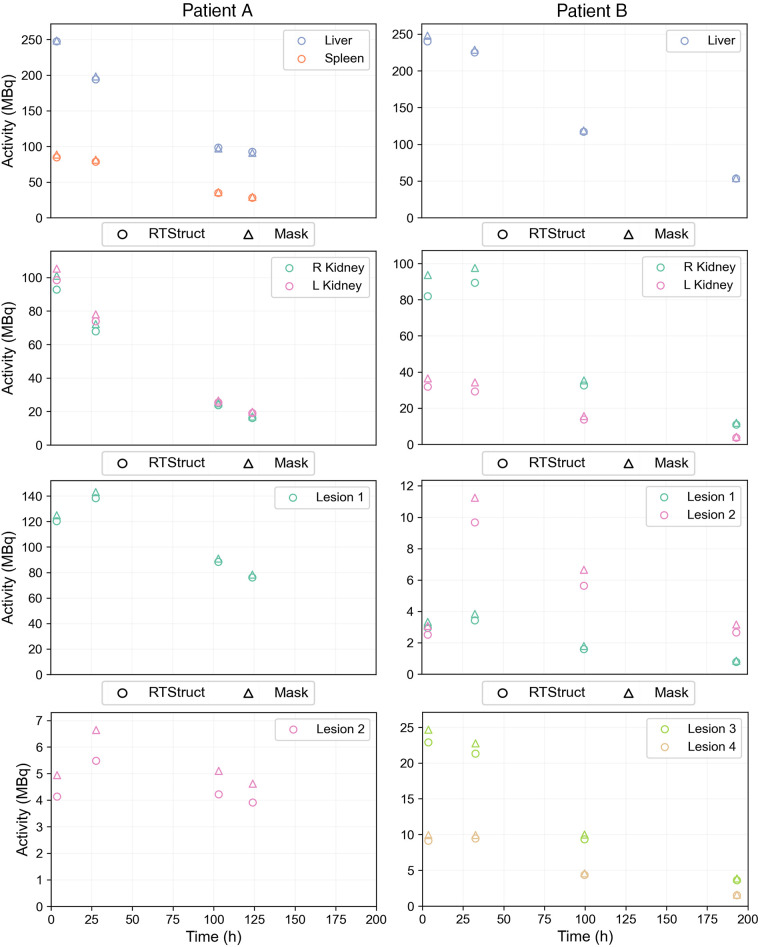
Scatterplots of time–activity data for all regions recreated (and used in baseline analysis) using VOIs in RTStruct and mask formats from task 4.

**FIGURE 5.**
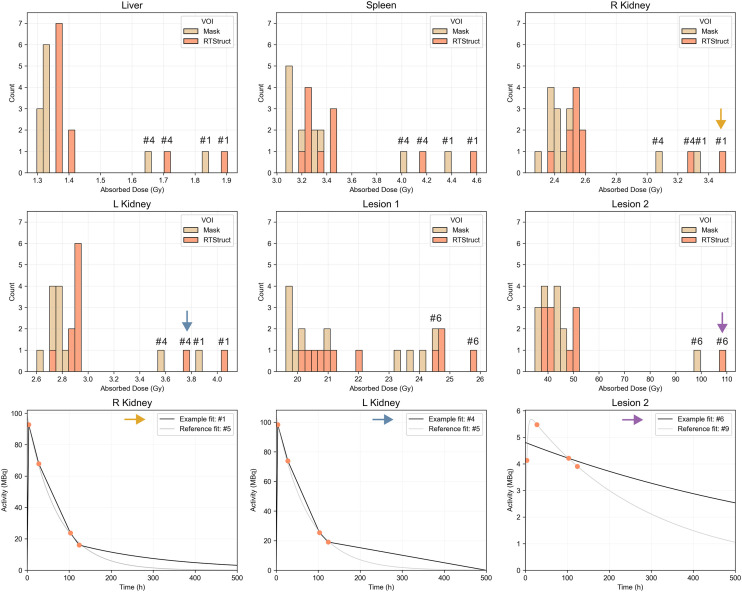
Patient A histograms of ADs calculated from baseline dataset (time–activity dataset obtained by authors from provided VOIs in RTStruct and mask formats), with fit numbers from Figure 1 highlighting methods yielding higher AD estimates. Methods consistently producing higher estimates, marked by arrows, are plotted on time–activity plots, together with reference methods: monoexponential (Fig. 1, method 5) for kidneys and biexponential (Fig. 1, method 9) for lesion.

**FIGURE 6.**
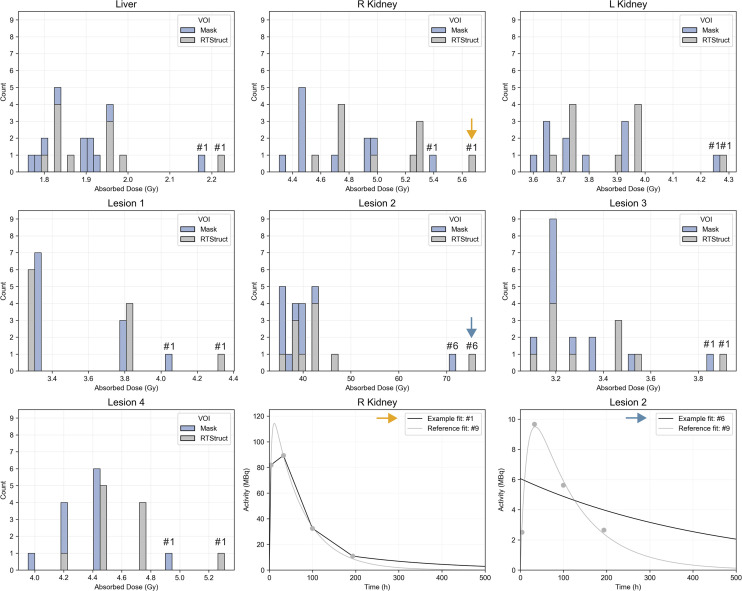
Patient B histograms of ADs calculated from baseline dataset (time–activity dataset obtained by authors from provided VOIs in RTStruct and mask formats), with fit numbers from Figure 1 highlighting methods yielding higher AD estimates. Methods consistently producing higher estimates, marked by arrows, are plotted on time–activity plots, together with biexponential model (Fig. 1, method 9) as reference.

Figure 4 presents time–activity data from task 4 (calculated using RTStructs and masks) that was used for the baseline analysis. The histograms in Figures 5 and 6 show AD distributions from the baseline analysis for all 11 methods at the organ level from Figure 1 for patients A and B, respectively. The annotated bars represent the fits from Figure 1 and highlight the approaches that resulted in the highest AD across all fits. The bottom rows present time–activity curves with these fits, illustrating how the approaches modeled the kinetics, alongside a reference method for comparison. For organs and lesions, method 1 most frequently resulted in the highest AD.

**FIGURE 7.**
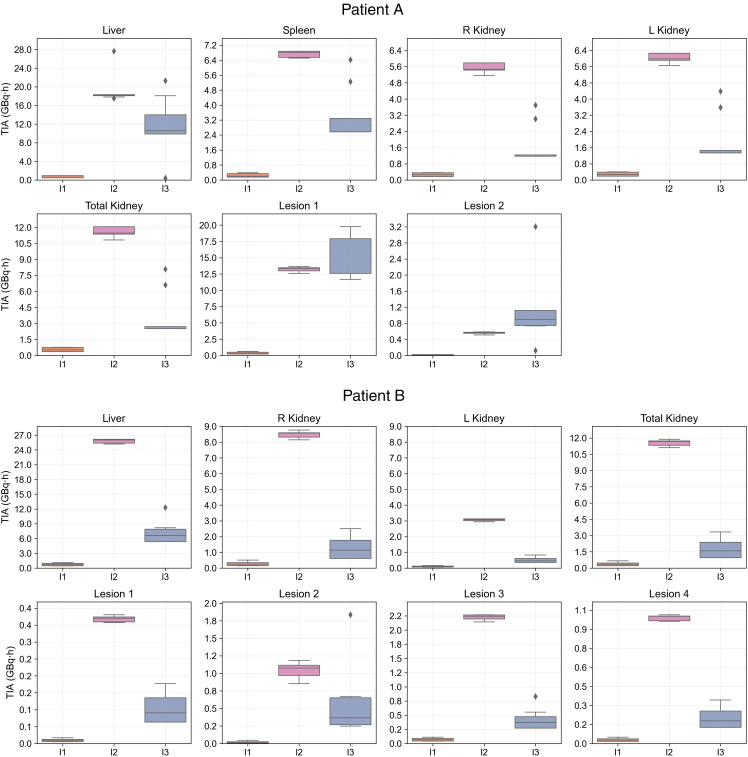
TIAs for each region in I1–I3 evaluated using baseline dataset for 11 fitting methods used by participants.

The Figure 7 box plots show each interval’s contribution to total TIA when grouping all 11 fitting methods. For method 8, in which activity was assumed to remain constant from 0 to the second time point, the I1 contribution was calculated as the area under the curve from 0 to the first time point to ensure comparability with the time intervals used in other methods. The highest variability comes from the integration on I3, which is not surprising, because it involves extrapolated time–activity curve data.

## DISCUSSION

Previous publications on factors affecting variability in the dosimetry calculations of the SNMMI ^177^Lu Dosimetry Challenge (5,13,18) showed combined variabilities from all elements of the dosimetry workflow. Here, we focused on analyzing variability in AD determination from time–activity data using submissions from tasks 4 and 5.

In the first step of this analysis, we identified the most used fitting methods for modeling different time–activity curve regions. Some models produced outliers and deserve some discussion. Specifically, the 4-parameter biexponential model (Eq. 4; Fig. 1, method 11) in some cases resulted in outliers in AD (Fig. 3). The fitting problem is ill-conditioned, because the number of parameters equals the number of time points. Therefore, it is not recommended to apply a 4-parameter model to fit 4 data points. Supplemental Figure 3A, where we replicated the plot of one of the participants, shows the model fit the data well, but it is not physiologically reasonable because of the high activity at time 0.

From Figure 4, we see that most organs had an almost instantaneous uptake; therefore, monoexponential fits were commonly used for organs (Fig. 1). However, some participants used monoexponential fits for lesions; for example, 3 participants used monoexponentials for lesion 2 of patient B, despite visual evidence (Fig. 4; Supplemental Fig. 3B) showing poor fit. Visual inspection is essential to ensure appropriate fitting functions (19).

The trapezoidal approach was frequently used to interpolate time–activity data, combined with several extrapolation methods for I3. When physical half-life was assumed (Fig. 1, method 1), AD results were higher than when the effective half-life was calculated (Figs. 3, 5, and 6). Although we recognize the benefits of simple models, they should be reserved for instances in which exponential models fail to fit the data adequately, and this was not the case for any region in the challenge dataset (Fig. 4). In addition, assuming physical decay after the last time point is a conservative approach, appropriate for radiation-protection purposes, but it may not be ideal for optimizing RPTs, because both overestimating and underestimating the AD can affect treatment optimization.

The second step of the analysis showed higher variability in ADs for lesions than for organs at risk, based on QCD differences between tasks and IQR of $\Delta \mathrm{AD}\;/\;{\bar{\mathrm{AD}}}_{\mathrm{task}5}$. This may be attributed to the more complex pharmacokinetics observed in lesions than in organs, suggesting that TIA and AD estimation for lesions could inherently have more variability because of uncertainties in time–activity data fitting and integration. Lesions typically showed an uptake phase followed by a washout phase (i.e., a biexponential behavior); a monoexponential function, which assumes instantaneous uptake, does not model this behavior. In addition, there was higher variability in the kidneys of patient B than in those of patient A. As before, the higher variability can be attributed to the slower uptake phase in patient B’s kidneys (Fig. 4). Similarly, among all lesions, ones with slower uptake, such as lesion 2 of patients A and B, had higher variability. For regions with slower uptake where the assumption of instantaneous uptake is invalid, a model that emphasizes robust curve fitting is essential, particularly during the slow washout phase, because it contributes most significantly to TIA (20). If data from multiple pharmacokinetic phases, both uptake and washout, are included, the model should account for these phases to prevent inaccuracies in later phases of the curve.

The observed higher variability in total kidney ADs than in individual kidney ADs (Fig. 2) may be explained by the likelihood that some participants added the ADs for each kidney separately, without accounting for differences in kidney mass. In addition, it can be caused by the estimation of the AD to 1 kidney without accounting for the mass of the combined kidneys in the dosimetry software. The accurate approach to combining the ADs of the separate kidneys is to perform the scaling of AD by the mass of each kidney to obtain a weighted average; an alternative approach is to ensure the mass of each kidney is used when calculated separately.

The paired analysis from Figure 3 shows that most outliers were observed with in-house fitting implementations. In addition, as noted in Supplemental Table 1, more outliers were reported for patient A than for patient B, potentially because of a failure to perform decay correction, which was required for patient A but not for patient B. The original spreadsheets for task 4 lacked activity at each time point, limiting our ability to verify whether decay correction was applied.

Step 3, conducted by the dosimetry expert, revealed a narrower AD range than that of the participant results. Outliers from participants’ analyses could not be reproduced using standard practices, even with the same fitting methods, suggesting these outliers stemmed from errors (e.g., software issues) or deviations from best practices, such as improper decay correction, rather than inherent variability in the fitting process.

This baseline analysis showed that simplified approaches, such as trapezoidal interpolation with physical or linear decay after the last time point (Fig. 1, methods 1 and 4), as well as models that do not apply additional constraints on the fit (Fig. 1, method 6), resulted in higher ADs in organs and lesions than with other models (Figs. 5 and 6). ADs calculated with RTStructs were higher than those calculated with binary masks, despite masks showing higher activity levels (Fig. 4), because RTStructs represented smaller volumes.

The variability in TIA calculation was highest in I3 (Fig. 7), emphasizing the importance of appropriate and consistent extrapolation after the last time point to decrease the variability in TIA and AD estimates. To mitigate errors from the contributions within this interval, the European Association of Nuclear Medicine guidelines recommend that the fractional contribution of the extrapolations from I3 to the TIA should be less than 20% (21). In a comparison of I3 results for patient A (last SPECT at 124 h after injection) and patient B (last SPECT at 193.3 h after injection), outliers in patient A’s data led to a TIA value double the median. This suggests that including later scans, as with patient B, could help mitigate such outliers. Variability in I1 was small because of its short duration and limited contribution to the total area under the curve, although assumptions during this interval still influenced overall TIA variability.

During the analysis, we identified several issues in participants’ submissions that affected activities, which serve as the input for pharmacokinetic modeling. These included improper decay correction and the inclusion of lesions in the assessment of healthy liver.

For patient A, images were decay-corrected to the injection time, whereas for patient B, they were corrected to the start of the SPECT scan. Despite instructions, some participants did not take this into account correctly, resulting in TIA overestimation ranging from 43% to 204%. This suggests that a quality-control check is needed to ensure that assumptions about decay correction are consistent with the data.

When estimating AD to healthy tissue for ^177^Lu toxicity predictions, it is essential to clearly define VOIs. Specifically, the uptake of activity by lesions located within the tissue (e.g., lesions in the liver) should not be included in calculating the total organ TIA (13); including this activity could significantly increase the whole-organ TIA and result in an overestimation of AD to healthy tissue. For the challenge data, the TIA for patient A’s liver, calculated with Equation 3, was 93.4% larger when lesions were included. Patient B, who had only 2 small liver lesions, showed a 6% increase. This highlights the need for agreement on the structures on which AD calculations are performed.

We acknowledge certain limitations in this study. First, the small number of patients (only 2) limits the generalizability of the findings, which are specific to ^177^Lu-DOTATATE and may not apply to other therapies. In addition, this study focused on variability in dosimetry results, rather than assessing their accuracy. Task 4 aimed to minimize the impact of segmentation-related variability. However, quantities calculated in VOIs differed when using RTStructs and binary masks, depending on how the software participants used calculated VOI quantities from RTStructs, which define VOIs by polygons. It does point out the need to harmonize not just VOI interchange formats but also how VOI quantities are calculated from them. Another limitation was the spreadsheet design for task 4, which did not request activities or fitting parameters, data that would have helped identify errors and issues. Although the baseline analysis was conducted by a single dosimetry expert, the results and methods were extensively discussed within the team to minimize potential errors. We recognize the limitation of relying on 1 analyst but believe this was mitigated through Dosimetry Challenge team discussions. Voxel-level fitting was not explored because of the larger number of complexities that must be addressed during its implementation. Voxel-level noise and the requirement for precise voxel-level registration can introduce significant variability, but it was a challenge to pursue it at this stage when participants were asked to report organ-level values (although they could have calculated them with voxelized approaches). However, the TIA map provided in task 5 was created using voxel-level fitting. We observed that this approach resulted in artifacts in some voxels because of alignment issues. Also, to decide on the best model, the Akaike Information Criterion was used instead of the corrected Akaike Information Criterion, despite the small amount of data. Lastly, in all tasks, many submissions lacked sufficient detail on the fitting methods, parameters, and results, making them difficult to interpret or replicate. We emphasize the importance of systematic and detailed reporting of dosimetry methods to ensure reproducibility and clarity in the field.

Nuclear medicine societies have been actively engaged in ongoing efforts to establish recommended best practices for dosimetry workflows (22). There is also substantial existing literature discussing the performance of time–activity data fitting methods and their impact on TIA and AD variability (6–8). This study highlights an ongoing need for improvement in adherence to current best practices in fitting and integration in clinical settings. Considering the outcomes of this work and the findings from these referenced studies, we have identified 4 critical areas in which the field could improve by establishing harmonized practices that are feasible even in busy clinical environments.

The first area, assess fit quality, would ensure proper fitting functions through visualization and knowledge of the shape of the time–activity curve for organs and lesions for the particular agent (15,19). Although quantitative metrics of goodness of fit should be used and reported, relying solely on metrics such as the coefficient of determination to accept or reject a fit would seem unwise. Some outliers observed in our analysis would have been avoided by following this recommendation (Supplemental Figure 3).

For the second area, optimize pharmacokinetic modeling, the use of fitting and exponential functions, rather than trapezoidal integration, should be preferred when they reasonably fit the data. This is primarily because of the need to perform extrapolation from the last time point to infinity. Use of a model that prioritizes robust curve fitting, particularly during the slow washout phase, contributes most significantly to dosimetry (20). If data from multiple pharmacokinetic phases, including both uptake and washout, are included, the model should account for these phases to prevent inaccuracies in later phases of the curve. There might be cases in which acquiring an extra imaging time point would improve the calculations (e.g., a lesion with the uptake phase would benefit from the collection of points that allow modeling of that phase).

With regard to the third area, carefully validate time–activity curve estimation software, most outliers observed in this study were associated with in-house software, which may lack the robustness or validation of commercial packages. It is crucial to benchmark all software, especially in-house tools, using well-established datasets before the software is applied to new data. We recommend using literature-based datasets, such as ground truth synthetic data (15) or challenge data from patient A in Supplemental Table 2, as gold standards to test the fitting and integration performance. Alternatively, new software can be validated by comparing its results with those from well-established software.

The fourth area, implement sanity checks, would incorporate sanity checks into the dosimetry workflow to ensure data are decay-corrected to scan time, input and output units are accurate, and results are presented systematically and clearly. For more on structured reporting, see Lassmann et al. (23) and Sgouros et al. (24).

## CONCLUSION

This study aimed to evaluate variability in ADs arising from different fitting and integration approaches reported by participants across different institutions. Kidneys of patient A showed low variability in AD due to the fitting step (<1%). Kidneys of patient B show higher variability (<10%), which is likely attributed to slower uptake in patient B’s kidneys. Lesions exhibited more variability in fitting than did kidneys (within 25%). The variability introduced by the fitting and integration step of the dosimetry workflow can be reduced by following our recommendations for standardization.

## DISCLOSURE

This work was partly supported by the SNMMI Value Initiative. Yuni Dewaraja acknowledges funding from National Cancer Institute (NCI) grant R01CA240706 for patient studies and resources made available by the University of Michigan Deep Blue Data Repository for data sharing. Eric Frey is a cofounder and part owner of Rapid, LLC; receives royalty income from GE HealthCare; and acknowledges support from grants R44CA213782 and R01CA240779 awarded by the NCI. Carlos Uribe acknowledges funding from Natural Science and Engineer Research Council discovery grant RGPIN-2021-02965. No other potential conflict of interest relevant to this article was reported.

## Notes

KEY POINTS **QUESTION:** What is the contribution of the TIA calculation step to the total variability in AD estimates in RPT when different fitting methods are used at different centers? **PERTINENT FINDINGS:** The contribution of the TIA calculation step to the total variability in AD value was found to be less than 10% for all healthy organs. It was generally larger for lesions, showing variability of less than 25%. **IMPLICATIONS FOR PATIENT CARE:** Analysis of the fitting and integration step of the dosimetry workflow for SNMMI ^177^Lu Dosimetry Challenge data has demonstrated that variability in ADs across different centers due to fitting methods for healthy organs was low.
